# Integrated therapy including acupuncture in a patient with long-term survival from spinal cord metastases of ALK-positive lung adenocarcinoma: a case report

**DOI:** 10.3389/fmed.2026.1815886

**Published:** 2026-06-23

**Authors:** Xiaojun Zhang, Guoping Ye, Wenzhen Chen, Jianwen Luo, Xiaoli Wu

**Affiliations:** 1 Department of Oncology, Xiamen Hospital of Traditional Chinese Medicine Affiliated to Fujian University of Traditional Chinese Medicine, Xiamen, China; 2 Department of Acupuncture and Moxibustion, Xiamen Hospital of Traditional Chinese Medicine Affiliated to Fujian University of Traditional Chinese Medicine, Xiamen, China

**Keywords:** acupuncture, case report, muscle strength, spinal cord metastasis, survival time

## Abstract

**Background:**

Lung cancer remains a leading cause of cancer mortality globally. Spinal cord metastases (SCMs) signify an advanced disease stage with a particularly dire prognosis, primarily due to severe neurological deficits and subsequent life-threatening complications. While modern systemic therapies can control tumor growth, they often inadequately address the critical issue of neurological recovery, which may be the key determinant of ultimate survival.

**Case presentation:**

A 47-years-old female with ALK-positive lung adenocarcinoma developed multiple SCMs. Following post-SCM systemic therapy, tumor burden decreased but limb strength deteriorated. Adjunctive acupuncture then led to improved muscle strength and quality of life. Overall survival from SCM diagnosis was over 9 months.

**Conclusion:**

This case highlights neurological recovery as a key goal in SCMs. Integrating acupuncture with standard therapy shows promise for improving function and outcomes, warranting further study.

## Background

Lung cancer remains a major global health burden. Spinal cord metastasis (SCM) represents a devastating complication, often causing severe neurological deficits in advanced disease. The prognosis is exceptionally poor, with a median overall survival typically between 2 and 3 months (1). Management is challenging due to patient frailty and a lack of standardized guidelines.

While surgical intervention (2) and radiotherapy (3) are often prioritized, many patients with multi-segment SCMs are not candidates for these localized approaches. Systemic drug therapy (chemotherapy and targeted agents) then becomes the mainstay, despite its limited efficacy against central nervous system metastases. This limitation is primarily attributed to the variable and often suboptimal penetration of the blood-brain barrier (BBB) by these agents, which can restrict their full therapeutic potential (4).

Critically, even when systemic therapy achieves measurable tumor reduction–as observed in our patient–the clinical benefits in terms of neurological recovery and survival are often disproportionately modest. This discrepancy suggests that tumor control alone is insufficient. SCMs initiate secondary injury cascades (e.g., inflammation, ischemia) that perpetuate neurological damage, leading to life-threatening complications such as infections and organ dysfunction, which are major drivers of mortality (5). Therefore, the devastating consequences of paralysis, rather than the tumor volume itself, frequently determine the outcome.

In this context, acupuncture, a key component of traditional Chinese medicine, is increasingly recognized as a valuable adjunct in integrative oncology and neurorehabilitation. While direct evidence in SCMs is limited due to disease rarity, its application in managing cancer-related symptoms (e.g., fatigue) and chemotherapy-induced peripheral neuropathy is supported by growing clinical evidence (6, 7). The proposed advantages of acupuncture include its multimodal action–potentially modulating neuroinflammation, improving local microcirculation, and enhancing neuroplasticity–which aligns with the need to address the secondary injury cascade in SCMs (8, 9). Furthermore, it is generally safe with minimal risk of interaction with systemic therapies and is relatively low-cost, making it a feasible adjunctive option for frail patients (10). These properties make acupuncture a promising, yet underexplored, strategy for targeting neural repair and functional preservation in SCMs.

Thus, there is an urgent need for adjunctive strategies that specifically target neural repair and mitigate these complications. We present a case managed by integrating standard anticancer therapy with acupuncture, aiming to address this unmet need.

## Case presentation

### Patient history and characteristics

The patient was a 45-years-old female with a past medical history notable only for the surgical excision of benign breast cysts a decade prior. She had no other significant comorbidities, no known drug allergies, and no family history of lung cancer or major genetic disorders in her first-degree relatives. She was a lifelong non-smoker.

### Diagnostic process

**FIGURE 1:**
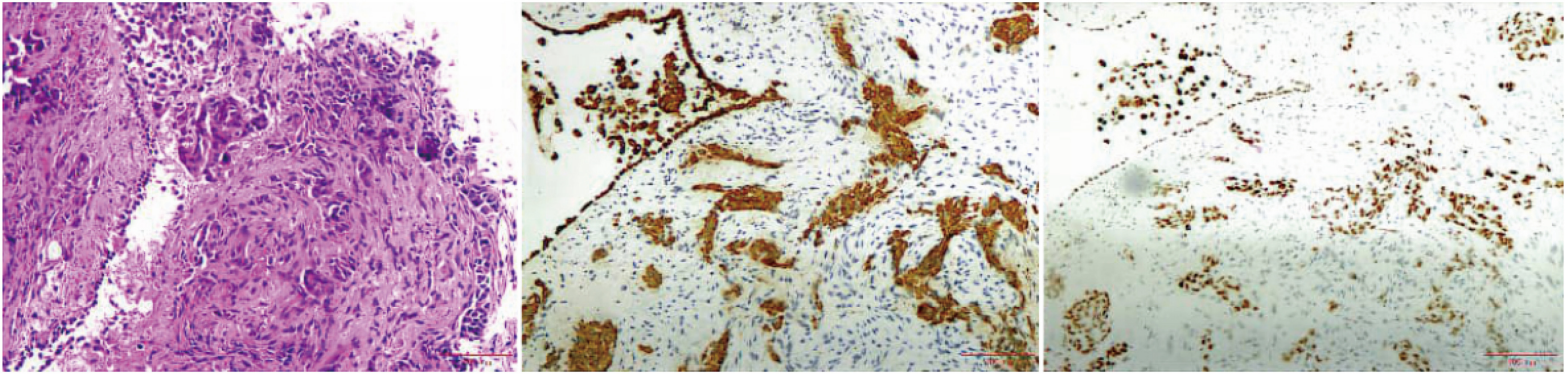
It shows her bronchoscopy biopsy pathology.

The patient presented to an external hospital in July 2017 with cough and dyspnea. As documented in the external hospital records, a chest CT scan revealed a right lung mass with mediastinal lymphadenopathy and pericardial effusion, raising a high suspicion of malignancy. A bronchoscopic biopsy of the lung lesion and a pericardiocentesis were performed for definitive diagnosis. Histopathology confirmed adenocarcinoma in both specimens (Figure 1), and subsequent molecular profiling identified an ALK gene rearrangement, confirming ALK-positive lung adenocarcinoma.

#### Diagnostic challenges and reasoning

The main challenge was obtaining sufficient tissue for both histological and molecular analysis. The combined procedure (bronchoscopy and pericardiocentesis) was used to maximize diagnostic yield and to determine the nature of the pericardial effusion. The conclusive pathology and molecular results rendered further differential diagnosis of the primary tumor unnecessary.

#### Initial differential diagnosis

Prior to biopsy, radiological findings warranted consideration of other malignancies (e.g., other lung cancer subtypes, metastasis from an occult primary) and, less likely, infectious or inflammatory causes of pericardial effusion.

#### Prognostic characteristics at diagnosis

The diagnosis of stage IV disease with multi-site metastases (pleural, pericardial) indicated a traditionally poor prognosis. Key negative factors included malignant pericardial effusion (suggesting advanced local invasion) and poor initial performance status. However, the identification of the ALK alteration was a positive predictive factor for targeted therapy response.

Following the diagnosis, the patient’s overall management, including the context for future integrative therapy, was guided by her primary lung cancer diagnosis and ALK-positive molecular profile. She received multiple lines of prior targeted therapy and radiotherapy (summarized in Table 1).

**TABLE 1:** Summary of prior systemic and local therapies administered before the diagnosis of spinal cord metastases (July 2017 – July 2022).

Time period	Systemic targeted therapy	Radiotherapy/surgical intervention
Jul – Oct 2017	Crizotinib	
Oct 2017 – May 2018	Traditional Chinese herbs (self-administered)	
May 2018 – May 2019	Brigatinib	
Jul 2019 – Jul 2020	Lorlatinib	
Jul 2020 – Mar 2021	Lorlatinib	Gamma Knife radiotherapy
Mar – May 2021	Alectinib + Bevacizumab	
May 2021		Ommaya reservoir implantation; resection of right cerebellar mass.
Jun – Nov 2021	Ensartinib	
Nov – Dec 2021	Alectinib + Temozolomide	Whole-brain radiotherapy
Dec 2021		Gamma Knife radiotherapy (×2 sessions)
Jan – Mar 2022		
Apr 2022	Ensartinib + Temozolomide	

On July 22, 2022, she presented with multiple spinal cord metastases (SCMs). Initial neurological examination revealed normal upper limb strength, bilateral lower limb strength of grade 4, and preserved ability to walk over 20 meters with assistance. Between July and September 2022, she completed three cycles of pemetrexed-cisplatin chemotherapy combined with anlotinib. A follow-up MRI demonstrated partial remission of the spinal metastases.

However, upon readmission for her fourth chemotherapy cycle in early October 2022, a significant neurological decline was noted: her proximal core and lower limb strength had decreased to grade 3 bilaterally, and she could no longer perform a hip bridge. This dissociation between continued radiographic tumor response and clinical functional deterioration prompted the initiation of adjunctive acupuncture on October 8, 2022.

The acupuncture was administered by one of the co-authors, a licensed attending physician with a Ph.D. in Acupuncture, ensuring expert delivery. The regimen was administered five times per week, with each 30-min session designed according to the principle of “Unblocking the Governor Vessel” integrated with modern neurorehabilitation theory. The standardized point selection targeted three key aspects: (1) regulating the Governor Vessel using Jiaji (EX-B2) points adjacent to the metastatic spinal level and Baihui (GV20); (2) strengthening the lower limbs via Biguan (ST31), Zusanli (ST36), Yanglingquan (GB34), Xuanzhong (GB39), Huantiao (GB30), and Chengfu (BL36); and (3) regulating systemic qi with Sanyinjiao (SP6) and Taichong (LR3). Sterile, single-use acupuncture needles of 0.30 mm × 40 mm were used for the trunk and buttock points, while 0.25 mm × 25 mm needles were used for the distal limb points. A standardized needling technique was applied: oblique insertion to 0.8–1.0 cun at the Jiaji points and perpendicular insertion to 1.2–1.3 cun at limb points, with manual manipulation to achieve deqi. Following manual stimulation to achieve deqi, electroacupuncture using a dense-disperse wave (2/15 Hz) was applied to paired points to elicit rhythmic, tolerable muscle contractions.

**FIGURE 2:**
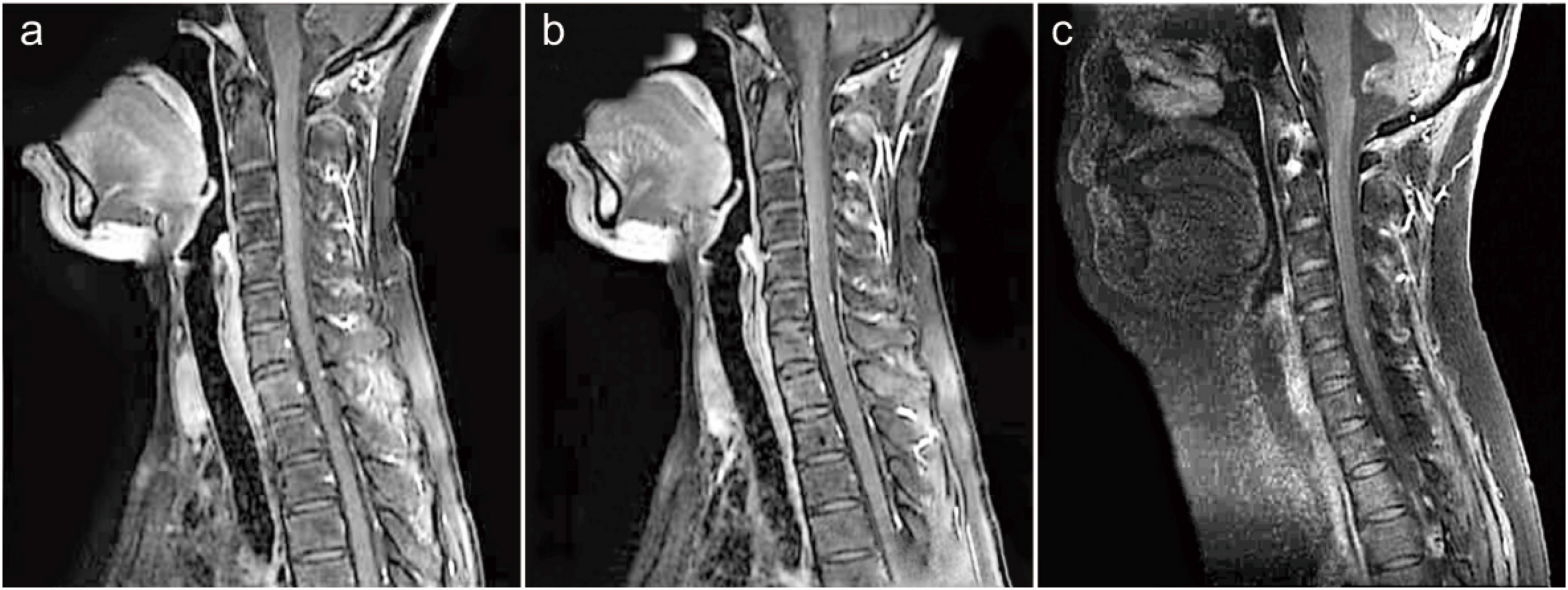
It shows that the lesion in the cervical spinal cord has decreased in size and density. **(a,b)** Is a magnetic resonance imaging taken in an external hospital in July 2022, and **(c)** is a magnetic resonance imaging taken in our hospital in October 2022.

**FIGURE 3:**
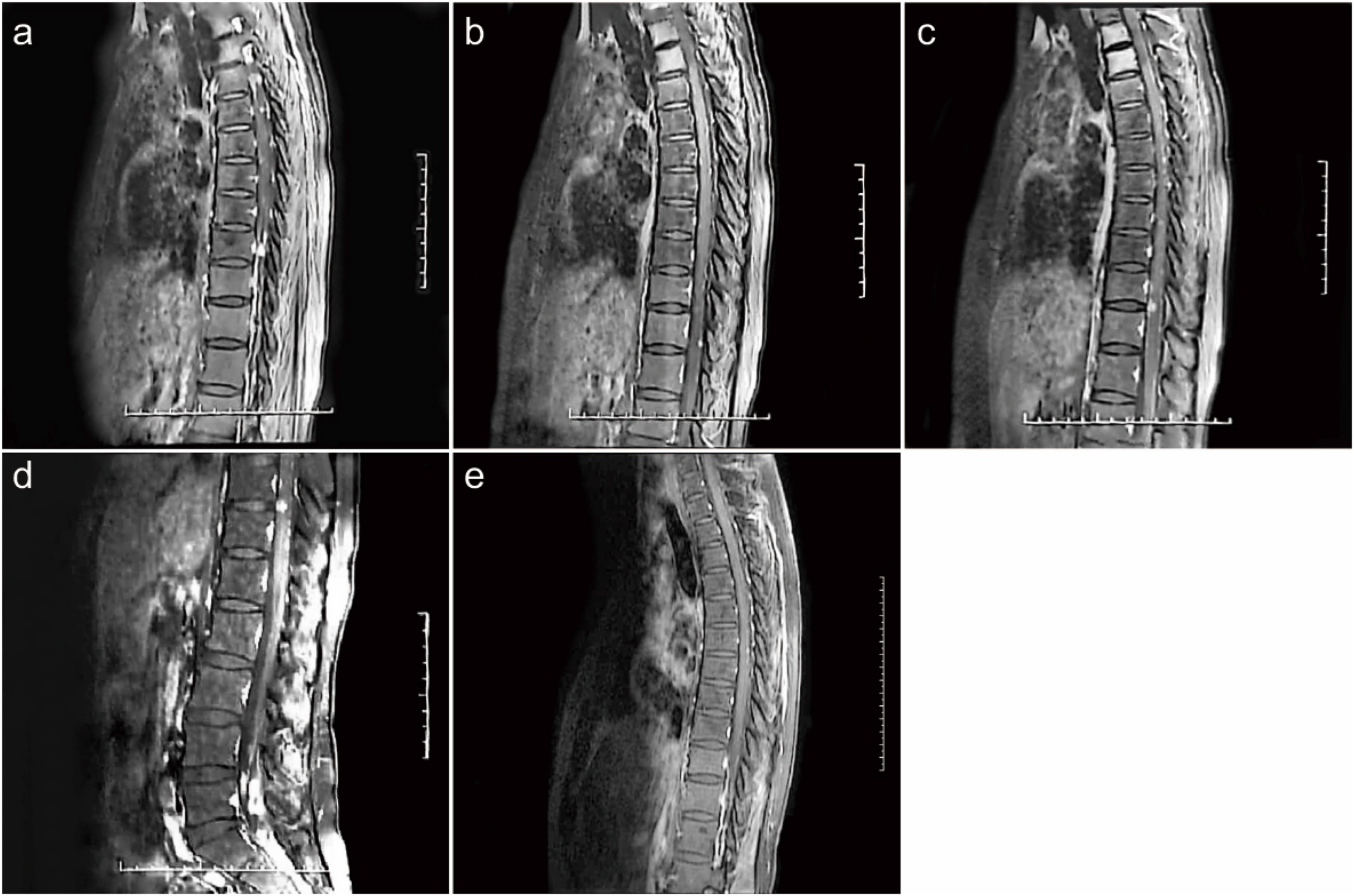
Magnetic resonance imaging (MRI) comparison of thoracic spinal cord metastases before and after treatment. **(a–d)** Baseline images (obtained from an external hospital in July 2022; photographs of the workstation screen). **(a–c)** Thoracic spine images showing multiple metastatic lesions. **(d)** Lumbar spine image, in which a prominent metastatic lesion in the lower thoracic segment is clearly visible. This image is included due to the limited availability of baseline images, to provide a clear baseline reference for comparison. **(e)** Follow-up image (acquired at our hospital in October 2022), showing marked reduction of the thoracic spinal cord metastases.

**FIGURE 4:**
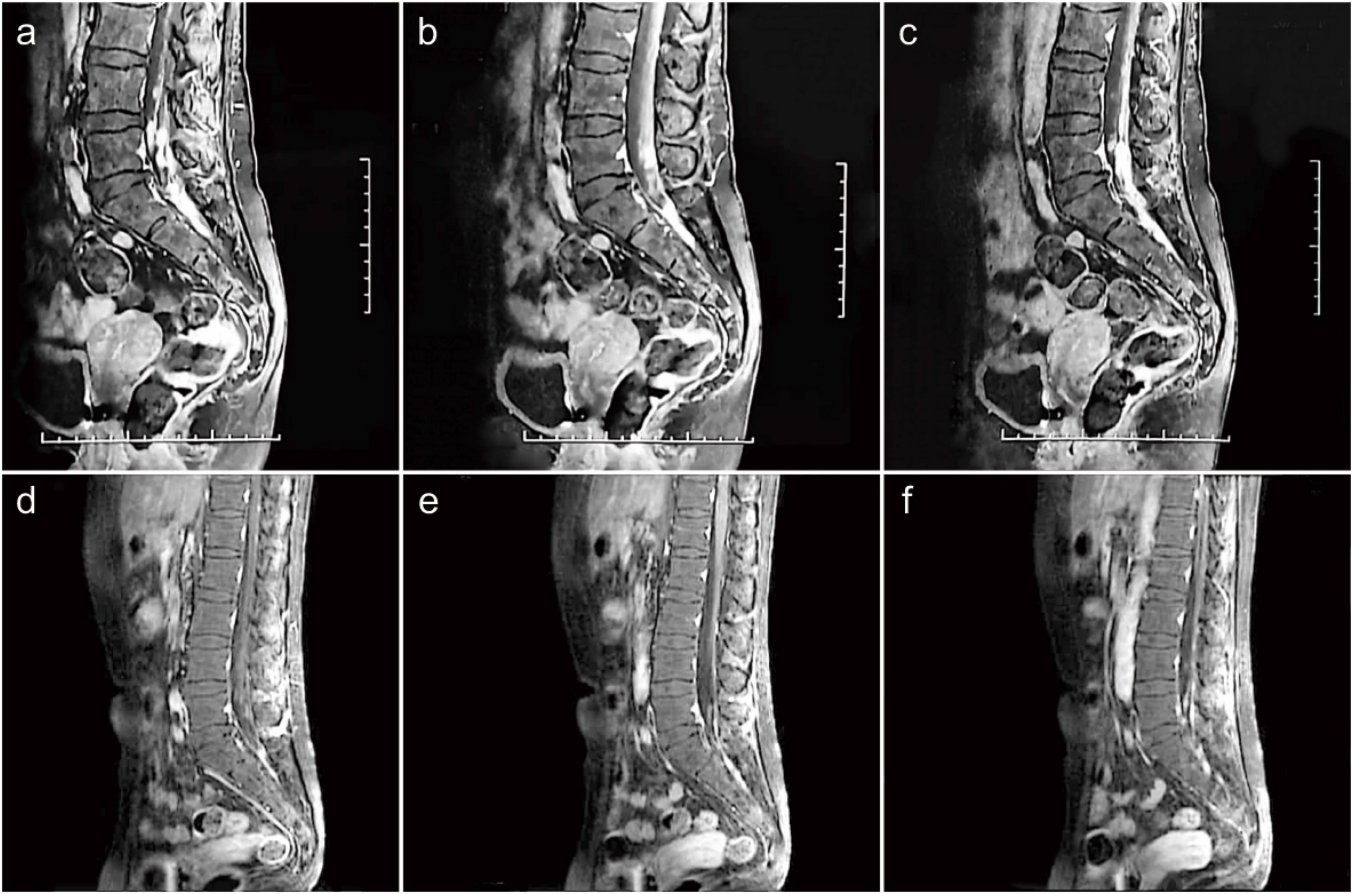
It shows that the lesions in the spinal cord of the lumbosacral region have significantly reduced and partially disappeared. **(a–c)** Shows MR images taken in an external hospital in July 2022, and **(d–f)** show those taken in our hospital in October 2022.

In late October 2022, the patient developed grade III bone marrow suppression. An MRI performed on October 31, 2022, confirmed further reduction in the spinal cord lesions (Figures 2–4). Given the ongoing radiographic response alongside manageable toxicity, she subsequently received two additional cycles of pemetrexed (without platinum) in November 2022, each followed by adjunctive acupuncture sessions. By mid-December 2022, her lower limb strength had recovered to grade 4, and she regained the ability to walk over 20 meters with assistance. Concurrently, she reported markedly improved mood and sleep quality, and expressed renewed hope regarding her treatment. Anlotinib was maintained until December 26, 2022.

Her subsequent hospitalization for SARS-CoV-2 infection in late December was complicated by viral pneumonia and secondary bacterial/fungal infections. Following discharge after supportive care, she remained bedridden with severe muscle weakness (grade 0–1+). While adjunctive acupuncture was continued, no further neurological recovery was observed following these cumulative insults. Beyond the motor deficits, the patient experienced profound psychological and emotional suffering as her physical condition declined. She reported symptoms of depressed mood, pervasive fear regarding her disease progression, severe insomnia, and at times, a loss of will to live. This multifaceted distress highlights the severe impact of metastatic spinal disease on a patient’s overall quality of life and mental well-being. The patient experienced recurrent pulmonary infections and died on April 10, 2023 (summarized in Table 2).

**TABLE 2:** Clinical course, treatment timeline, neurological status, and key imaging findings following the diagnosis of spinal cord metastases.

Time period	Systemic therapy (targeted/ chemotherapy)	Local/supportive therapy and major events	Neurological status and symptoms	Imaging follow-up
Jul 2017 – Jul 2022	Multiple lines of prior TKIs (see Table 1)	Multiple radiosurgical procedures (see Table 1)	No SCM-related symptoms documented	Not applicable
22 Jul 2022		Presented with multiple SCMs	Baseline: Lower limb muscle strength: Medical Research Council (MRC) grade 4; able to walk > 20 m with assistance.	Baseline MRI (Figure 2)
Jul – Sep 2022	Anlotinib + Pemetrexed/ Cisplatin (3 cycles)	Gamma Knife radiosurgery (20 Jul 2022)	Remained stable.	Sep 2022 MRI: Partial remission of spinal metastases.
Oct 2022	Anlotinib + Pemetrexed/ Cisplatin (4th cycle)		Significant deterioration: Lower limb strength declined to MRC grade 3 bilaterally; inability to perform a hip bridge.	
Starting 8 Oct 2022		Adjunctive acupuncture initiated (5 sessions/week).		
Late Oct 2022		Grade III myelosuppression occurred.		
31 Oct 2022				MRI: Spinal lesions showed further reduction (Figures 2–4).
Nov 2022	Anlotinib + Pemetrexed monotherapy (2 cycles)	Acupuncture continued.	Gradual improvement.	
Mid-Dec 2022	Anlotinib maintained (until 26 Dec)	Acupuncture continued.	Recovery: Lower limb strength returned to MRC grade 4; regained assisted walking ability (>20 m).	
Late Dec 2022		Hospitalized for SARS-CoV-2 infection, complicated by pneumonia and secondary bacterial/fungal infections; received antibiotics/antifungals.	Acute decline: Bedridden with severe weakness (MRC grade 0–1+).	
Jan – Feb 2023	Anlotinib	Acupuncture continued (no further functional recovery observed).	Remained bedridden with severe muscle weakness.	
10 Apr 2023		Death.		

## Discussion

The management of spinal cord metastases (SCMs) remains inconsistent and poorly standardized, leading to divergent clinical outcomes (2). Unlike metastases in other sites, central nervous system involvement entails distinct biological challenges. Spinal cord injury (SCI) in the oncological setting involves both primary and secondary injury mechanisms, which frequently result in progressive neurological decline, paraplegia, and life-threatening complications.

In this case, tumor shrinkage after therapy coincided with declining muscle strength. This clinical-radiographic dissociation suggests the potential presence of treatment-related toxicity, and such toxicity (e.g., from cisplatin) may offset tumor control (11). It highlights the RANO principle of independent neurological assessment (12). Thus, SCMs strategies must equally prioritize antitumor efficacy and neurological preservation.

The pathological cascade following SCI involves multiple processes that hinder recovery, including oxidative stress (13, 14), neuronal apoptosis (15), neuroinflammation (16), vascular compromise (17), and glial scar formation (18). While conventional rehabilitation optimizes residual function (19), it does not directly promote neural repair. This therapeutic gap motivated the integration of acupuncture into our patient’s regimen.

Our acupuncture protocol was designed to “Unblock the Governor Vessel,” combining local Jiaji points (para-spinal points) with distal points (e.g., Zusanli, ST36) to regulate qi and blood flow and strengthen the limbs. This integrative approach is supported by a growing body of preclinical evidence demonstrating that acupuncture and electroacupuncture can modulate multiple key pathways following spinal cord compromise. Specifically, it can reduce oxidative stress (20, 21), protect nerves by attenuating the apoptosis of neurons and oligodendrocytes (22), and mediate neuroprotective effects by inhibiting neuroinflammation and microglial activation (8, 23, 24). Furthermore, acupuncture can regulate microcirculation to alleviate neurological dysfunction (9, 25), mitigate the formation of glial scars–a major barrier to regeneration–by modulating astrocyte activity (26, 27), and promote repair by inducing the proliferation and differentiation of neural stem cells (28–30). In line with these mechanisms, we applied electroacupuncture with a dense-disperse wave (2/15 Hz) to provide rhythmic neuromuscular stimulation.

In this case, acupuncture was associated with temporary recovery of muscle strength and quality of life. Its delayed initiation reflects a common clinical oversight regarding the urgency of neurological rehabilitation. The patient’s multi-line therapy, disease progression, and COVID-19 infection (31) placed cumulative strain on her nervous system, which may have limited sustained recovery.

The limitations of acupuncture in this setting are threefold. First, it lacks direct antitumor activity, aiming only to mitigate neurological damage. Second, its delayed initiation after neurological decline likely missed the optimal neuroprotective window, limiting its potential benefit. Finally, its effect was inevitably constrained by severe concurrent factors, including progressive tumor burden, radiation-induced neurotoxicity, and COVID-19 infection. Future studies should first validate its benefit in larger cohorts, then determine if earlier integration at initial spinal involvement can better preserve function and quality of life.

When viewed against the typical median survival of 2–3 months for patients with multi-segmental SCMs (1), the survival period observed in this case may represent a clinically meaningful extension. Although the primary goal was neurological support, any potential extension of the survival window–even by several months in advanced disease–may provide a critical opportunity to bridge to subsequent therapies. Thus, the preservation of neurological function through adjunctive means like acupuncture could indirectly influence survival outcomes by maintaining a patient’s eligibility for further treatment.

Beyond enabling continued therapy, preserving neurological function is equally critical for mitigating the severe psychological sequelae of functional loss. This case illustrates that the patient’s psychological distress–including fear, depressed mood, and loss of will to live–was directly tied to her neurological decline. This underscores the critical importance of interventions that improve neurological function. Here, acupuncture aimed at “Unblocking the Governor Vessel” targeted this need. The observed temporary muscle strength recovery was thus vital, as by alleviating the core disability, it likely also mitigated the associated fear and suffering, thereby supporting her overall will to live.

## Conclusion

This case underscores the imperative for early integration of functional support strategies upon the diagnosis of spinal cord metastases (SCMs). Given the rapid establishment of secondary injury cascades, initiating adjunctive modalities such as acupuncture alongside standard antitumor therapy for SCMs may help mitigate neurological deterioration by modulating these pathological processes. While controlling the metastatic lesion remains paramount, a parallel and immediate focus on neurological preservation from the outset is essential to improve outcomes. Acupuncture represents a mechanistically grounded, supportive approach within this integrated strategy, meriting future prospective studies to evaluate its impact on functional preservation and quality of life.

## Data Availability

The original contributions presented in this study are included in this article/supplementary material, further inquiries can be directed to the corresponding authors.
